# Seasonal variation in vascular dehydration risk: insights from the Kobe Orthopedic and Biomedical Epidemiologic (KOBE) study

**DOI:** 10.1265/ehpm.24-00132

**Published:** 2024-11-02

**Authors:** Tomofumi Nishikawa, Naomi Miyamatsu, Aya Higashiyama, Yoshimi Kubota, Yoko Nishida, Takumi Hirata, Aya Hirata, Junji Miyazaki, Daisuke Sugiyama, Kazuyo Kuwabara, Sachimi Kubo, Yoshihiro Miyamoto, Tomonori Okamura

**Affiliations:** 1 Faculty of Health Science, Kyoto Koka Women’s University, Kyoto, Japan; 2 Department of Clinical Nursing, Shiga University of Medical Science, Shiga, Japan; 3 Department of Hygiene, Wakayama Medical University, Wakayama, Japan; 4 Department of Environmental and Preventive Medicine, School of Medicine, Hyogo Medical University, Hyogo, Japan; 5 Osaka Institute of Public Health, Osaka, Japan; 6 Human Care Research Team, Tokyo Metropolitan Institute for Geriatrics and Gerontology, Tokyo, Japan; 7 Department of Preventive Medicine and Public Health, School of Medicine, Keio University, Tokyo, Japan; 8 Faculty of Nursing and Medical Care, Keio University, Tokyo, Japan; 9 Department of Nutrition and Food Sciences, Tezukayama Gakuin University, Osaka, Japan; 10 Open Innovation Center, National Cerebral and Cardiovascular Center, Osaka, Japan; 11 Foundation for Biomedical Research and Innovation, Hyogo, Japan

**Keywords:** Dehydration, Seasons, Serum osmolarity, Hematocrit, Cross-sectional study

## Abstract

**Background:**

Dehydration, a risk factor for ischemic cerebrovascular diseases, is common in summer; however, the incidence of ischemic diseases is not necessarily higher in summer. Therefore, this study aimed to clarify the relationships between serum osmolarity, hematocrit, daily non-alcohol drink (NAD) intake and factors such as season and age as risk factors for dehydration.

**Method:**

Participants (703 women and 306 men) in the follow-up survey, in 2012 and 2013, of the Kobe Orthopedic and Biomedical Epidemiologic (KOBE) Study, consisting of healthy individuals living in Kobe, Japan, were categorized into two groups based on the examination month: the warmer and colder seasons. Multivariate analyses were conducted to examine disparities in serum osmolarity, hematocrit, and NAD intake between these two groups.

**Results:**

The colder season was found to be negatively correlated with serum osmolarity and NAD intake, but positively correlated with hematocrit, even after adjusting for relevant factors. Age was independently associated with serum osmolarity, but not with hematocrit and NAD intake.

**Conclusions:**

This study suggests that intra-vascular volume depletion is more likely in the colder season despite lower serum osmolarity compared to the warmer season. Age-related increases in serum osmolarity without a corresponding rise in water intake may contribute to this. These findings support the importance of addressing dehydration in the colder season, particularly in older adults.

## Introduction

Dehydration is considered a risk factor for ischemic diseases [1, 2]. However, it is not necessarily the case that ischemic diseases are more prevalent in summer [3], a season often associated with higher levels of dehydration. Many major cities in Japan belong to the temperate climate zone, experiencing distinct seasonal variations. During summer, temperatures often exceed 30 degrees Celsius with high humidity. In contrast, winter temperatures can fall below freezing, and the air tends to be dry [4]. The occurrence of ischemic stroke has been reported to rise with decreasing temperature [5, 6], and incidence rates of stroke are highest in winter and lowest in summer in Japan [7]. Extra mortality due to ischemic disease is also higher in winter than in summer in Japan [8], as well as in Europe [9], although it may depend on the regional climate [10]. This phenomenon is induced by fluctuations in environmental temperature, which in turn influence the physiological state of the organism, closely associated with ischemic diseases [9, 11, 12]. For instance, blood pressure [13, 14], the occurrence of atrial fibrillation [15, 16], and plasma fibrinogen concentration increase in winter [11]. On the other hand, dehydration, which is also one of essential causes of ischemic diseases, is more likely to occur in summer, and this discrepancy has not been clearly understood.

There is no universally recognized definition for dehydration. However, in clinical terms, it was defined as a deficiency of total body water [17]. In the literature, scientists have referred to the hydration status as either mild-dehydration, insufficient hydration, suboptimal hydration, pre-dehydration, or underhydration [18]. While there are several methods to assess hydration status in humans, no clear best practice guidelines exist for the assessment of hydration. Serum osmolarity and hematocrit (Ht) are valuable indicators for assessing dehydration from different perspectives. Serum osmolarity is used as a definitive diagnosis (reference standard) for water-loss dehydration, as serum and intracellular osmolarity are so central to body fluid control that they act as a trigger to both thirst and renal conservation of fluid [19]. On the other hand, while it is believed that there are no clear-cut standards for the threshold of Ht for dehydration, Ht can be influenced by intra-vascular volume depletion [20], which refers to a condition with a decrease in blood plasma volume without a corresponding reduction in the number of red blood cells. In addition, Ht is also thought to be a principal determinant of blood viscosity [21]. The decrease in blood plasma volume can cause vascular collapse [22], and aggregation of red blood cells can increase blood viscosity [23]; these factors are thought to be one of the primary causes of ischemic diseases. For example, Ht level of 51% or higher in men and 48% or higher in women is generally considered suspicious for polycythemia, and when dehydration is the cause, it is referred to as relative polycythemia [24]. In polycythemia, individuals are at an increased risk of thrombosis and arterial occlusion [25].

The drastic seasonal variation can lead to different health risks; however, specific prevalence data on dehydration in Japan is limited [12, 26]. In Japan, Tanaka et al. analyzed health checkup data from 903 individuals aged 65 and older, divided into four seasons, and reported that plasma osmolality was highest in the spring [12]. Thus, Japan’s aging population, which is more prone to dehydration due to physiological changes and chronic conditions, underscores the need for seasonal studies. Contrary, in a study that investigated serum osmolality before and after work in sixty male foresters (average age 32.3 years) who spend most of their work outdoors, it was reported that serum osmolarity was higher during colder seasons [27]. These research results suggest that hydration status changes with the seasons and age. However, there is limited research on this topic. Therefore, the present study aimed to clarify the relationships between serum osmolarity, hematocrit, daily non-alcohol drink (NAD) intake and factors such as season and age as risk factors for dehydration, using multivariate analyses, in relatively older general residents.

## Methods

### 1. Study population

We performed a cross-sectional study using data obtained from the first follow-up survey in the Kobe Orthopedic and Biomedical Epidemiological study (KOBE study), which is a population-based prospective cohort study of risk factors for cardiovascular disease or worsening of quality of life in Kobe, a major urban area located in west Japan where there is a clear difference in temperature depending on the season [28, 29]. For instance, the monthly mean temperatures in 2012 (annual average temperature 16.6 °C) are as follows: in January 5.7 °C, in February 5.2 °C, in March 9.1 °C, in April 15.2 °C, in May 19.4 °C, in June 23.0 °C, in July 27.2 °C, in August 29.3 °C, in September 26.2 °C, in October 19.8 °C, in November 12.7 °C, and in December 6.8 °C [4]. The study participants were volunteers aged 40 to 74 years who were residents of Kobe; the participants had to meet the following criteria: 1) not currently on medications for hypertension, dyslipidemia, and diabetes mellitus; and 2) no history of cardiovascular diseases and cancer. The KOBE study commenced in 2010, and during the baseline survey conducted from July 2010 to December 2011, 1,117 subjects (776 women and 341 men) participated. Follow-up surveys were conducted biennially after the baseline survey, as previously reported [29–33]. Among 1,117 subjects who participated in the first follow-up survey in 2012 and 2013, 76 didn’t express their will to participate in the first follow-up survey, 8 stopped participation, 8 died between the baseline survey and the first follow-up survey, 15 had missing information (11 didn’t attend the face-to-face interviews as well as on-site tests, 4 didn’t complete the blood test) and 1 didn’t take urine test; thus the data of 1009 (703 women and 306 men) were used for the following analyses. Among them, the sex ratios (women/men) for each surveyed month were as follows: January (59/16), February (84/44), March (55/11), May (60/30), June (50/12), July (61/27), September (76/54), October (46/25), November (120/33), and December (92/54). Thus, 441 (293 women and 148 men) participants attended the surveys between May and October (warmer season), and 568 (410 women and 158 men) participated in the surveys between November and December (colder season).

### 2. Data collection for characteristic

Characteristics were collected during the follow-up survey, which occurred 2 years after the baseline survey. The data collection procedures were consistent with those detailed in our prior studies [29–32]. These surveys were conducted every month except for April and August, and each participant was randomly divided into one of these months. Each subject completed a self-reported questionnaire to assess past medical history and life habits, including current smoker, current drinker, sleeping time, walking habit, and living alone. Evaluation of non-alcoholic and alcoholic drink intake was detailed in our prior studies [1]. Height and body weight was measured with patients wearing socks and light clothing, and body mass index (BMI) was calculated by dividing weight in kilograms by the squared height in meters. The blood pressure was measured twice in each participant after a five-minute rest using an automatic sphygmomanometer (Nihon Colin, BP-103iII), and the mean value for each participant was recorded. All blood samples were obtained in the morning after fasting for at least 10 hours, and blood samples were tested by one commissioned clinical laboratory center (SRL Inc., Tokyo, Japan). Blood glucose, blood urea nitrogen, serum sodium, serum potassium, and serum chloride were measured. Serum osmolarity (mOsm/L) was calculated by Worthley’s formula: 2 (serum sodium (mEq/L)) + (blood urea nitrogen (mg/dL))/2.8 + (glucose (mg/dL))/18 [34].

### 3. Statistical analysis

Continuous variables were analyzed using the Student t-test, while categorical variables were assessed with the chi-square test. Multivariate linear regression analysis was employed to evaluate an independent association of seasons (colder to warmer) with serum osmolarity, adjusting for sex, age, height, body weight, hematocrit (%), fasting blood glucose, NAD intake, hypertension (systolic blood pressure ≧140 mmHg, diastolic blood pressure ≧90 mmHg, or taking antihypertensive), dyslipidemia (LDL ≧140 mg/dL, triglyceride ≧150 mg/dL, HDL <40 mg/dL or taking medication for dyslipidemia), diabetes (fasting glucose >= 126 mg/dL, HbA1c >= 6.5 or taking medication for diabetes), current drinker, current smoker, sleeping hours, walking habit (≧2 days/a week, for ≧30 minutes) and living alone. In the same way, independent association of seasons (colder to warmer) with hematocrit was evaluated adjusting for the relevant factors, including serum osmolarity instead of hematocrit. All significance tests were two-tailed, and p < 0.05 was considered significant in all analyses. All statistical analyses were performed with IBM SPSS Statistics for Windows version 25 (IBM Corp., Armonk, NY).

### 4. Ethics approval and consent to participate

This study was conducted according to the guidelines laid down in the Declaration of Helsinki, and all procedures involving research study participants were approved by the Ethics Committees of the Institute of Biomedical Research and Innovation (Committee approval number: 11-12) in 2012 and Kyoto Koka Women’s University (Committee approval number: 012) in 2013. Written informed consent was obtained from all patients. Written informed consent was obtained from all participants.

## Results

Participant characteristics, stratified by sex and the two seasons, warmer and colder seasons, are presented in Table 1. The ratios of the participants in each season (warmer season/colder season) by age group were 35/62 under 50 years old (y.o.), 77/126 between 50 and 60 y.o., 123/170 between 60 and 70 y.o. and 58/52 over 70 y.o. in women, and 14/17 under 50 y.o., 22/36 between 50 and 60 y.o., 57/71 between 60 and 70 y.o. and 55/34 over 70 y.o. in men. Non-alcohol drink (NAD) intake and serum osmolarity were significantly or relatively higher in the warmer season compared to the colder season. When dehydration was defined as a serum osmolarity of 300 mOsm/L or higher [35], the proportion of dehydration was 22.7% in the warmer season and 17.2% in the colder season. When limited to participants aged 65 and older, the proportion of dehydration was 30.7% in the warmer season and 23.6% in the colder season, with an overall prevalence of 27.2%. At the same time, hematocrit was significantly lower in the warmer season than in the colder season in both sexes. No seasonal fluctuation was observed in systolic and diastolic blood pressure.

**Table 1:** Demographic Characteristics

**Women**							

	**Overall (Women)**		**Surveyed month**				**p value**

			**Warmer season**		**Colder season**		
Number of participants	703	-	293	-	410	-	
Age (y.o.)	60.3 ± 8.5		61.4 ± 8.6		59.6 ± 8.4		0.005
BodyWeight (Kg)	51.0 ± 7.3		50.7 ± 7.3		51.2 ± 7.4		n.s.
Height (cm)	155.7 ± 5.4		155.4 ± 5.6		155.8 ± 5.3		n.s.
BMI (Kg/m^2)	21.0 ± 2.8		21.0 ± 2.8		21.1 ± 2.8		n.s.
SBP	111.4 ± 15.9		112.4 ± 16.3		110.7 ± 15.6		n.s.
DBP	68.0 ± 10.2		68.1 ± 10.2		68.0 ± 10.2		n.s.
Ht. (%)	40.4 ± 2.7		40.1 ± 2.7		40.6 ± 2.7		0.019
Fasting blood glucose (mg/dL)	87.9 ± 8.2		87.8 ± 7.4		88.0 ± 8.7		n.s.
Sodium (mEq/L)	143.53	1.6	143.7	1.6	143.4	1.6	0.004
Chloride (mEq/L)	104.3	1.8	104.9	1.7	103.9	1.7	<0.001
Potassium (mEq/L)	4.3	0.3	4.3	0.3	4.3	0.3	n.s.
BUN (mg/dL)	14.1	3.3	14.2	3.4	14.0	3.3	n.s.
Serum Osmorality (mOsm/L)	297.0 ± 3.7		297.4 ± 3.7		296.6 ± 3.6		0.006
NAD intake (mL/day)	1768.3 ± 708.6		1940.0 ± 775.6		1645.5 ± 629.2		<0.001
Hypertension	68	9.7%	31	10.6%	37	9.0%	n.s.
Dyslipidemia	344	48.9%	144	49.1%	200	48.8%	n.s.
Diabetes	5	0.7%	1	0.3%	4	1.0%	n.s.
Lifestyles							
Current drinker	251	35.7%	97	33.1%	154	37.6%	n.s.
Current smoker	10	1.4%	4	1.4%	6	1.5%	n.s.
Sleeping hours	6.2 ± 1.0		6.3 ± 1.1		6.2 ± 1.0		n.s.
Walking habit	86	12.2%	36	12.3%	50	12.2%	n.s.
Living alone	17	2.4%	9	3.1%	8	2.0%	n.s.

**Men**							

	**Overall (Men)**		**Surveyed month**				**p value**

			**Warmer season**		**Colder season**		
Number of participants	306	-	148	-	158	-	
Age (y.o.)	63.1 ± 8.6		64.3 ± 8.4		62.0 ± 8.6		0.016
BodyWeight (Kg)	63.9 ± 9.1		63.4 ± 9.4		64.4 ± 8.8		n.s.
Height (cm)	167.3 ± 5.9		167.6 ± 5.8		167.1 ± 5.9		n.s.
BMI (Kg/m^2)	22.8 ± 2.6		22.5 ± 2.6		23.0 ± 2.6		n.s.
SBP (mmHg)	118.4 ± 15.7		117.2 ± 15.5		119.6 ± 15.7		n.s.
DBP (mmHg)	75.6 ± 9.5		74.6 ± 9.5		76.5 ± 9.4		n.s.
Ht. (%)	44.5 ± 3.3		43.8 ± 3.5		45.1 ± 2.9		0.001
Fasting blood glucose (mg/dL)	94.3 ± 12.6		93.2 ± 11.3		95.3 ± 13.6		n.s.
Sodium (mEq/L)	143.1 ± 1.5		143.3 ± 1.5		142.9 ± 1.5		0.029
Chloride (mEq/L)	103.8 ± 1.9		104.4 ± 1.8		103.3 ± 1.9		<0.001
Potassium (mEq/L)	4.3 ± 0.3		4.3 ± 0.3		4.4 ± 0.3		n.s.
BUN (mg/dL)	14.9 ± 3.1		15.0 ± 3.2		14.8 ± 3.0		n.s.
Serum Osmorality (mOsm/L)	296.8 ± 3.3		297.2 ± 3.4		296.5 ± 3.1		n.s.
NAD intake (mL/day)	1603.5 ± 612.8		1783.1 ± 559.4		1435.2 ± 614.4		<0.001
Hypertension	61	19.9%	31	20.9%	30	19.0%	n.s.
Dyslipidemia	120	39.2%	53	35.8%	67	42.4%	n.s.
Diabetes	16	5.2%	6	4.1%	10	6.3%	n.s.
Lifestyles							
Current drinker	234	76.5%	112	75.7%	122	77.2%	n.s.
Current smoker	33	10.8%	14	9.5%	19	12.0%	n.s.
Sleeping hours	6.2 ± 1.0		6.3 ± 1.1		6.2 ± 1.0		n.s.
Walking habit	109	35.6%	48	32.4%	61	38.6%	n.s.
Living alone	17	5.6%	9	6.1%	8	5.1%	n.s.

Continuous data was shown in the mean ± SD. Categorical data was is shown as number and %, and analyzed using the χ2 test. NAD; non-alcohol drink. BMI; body mass index. SBP; systolic blood pressure, DBP; diastolic blood pressure, BUN; blood urea nitrogen, Walking habit; >=2 days/a week, for >=30 min. n.s.; not significant (p >= 0.05). Warmer seasons; from May to October, Colder seasons; from November to April.

**Fig. 1:**
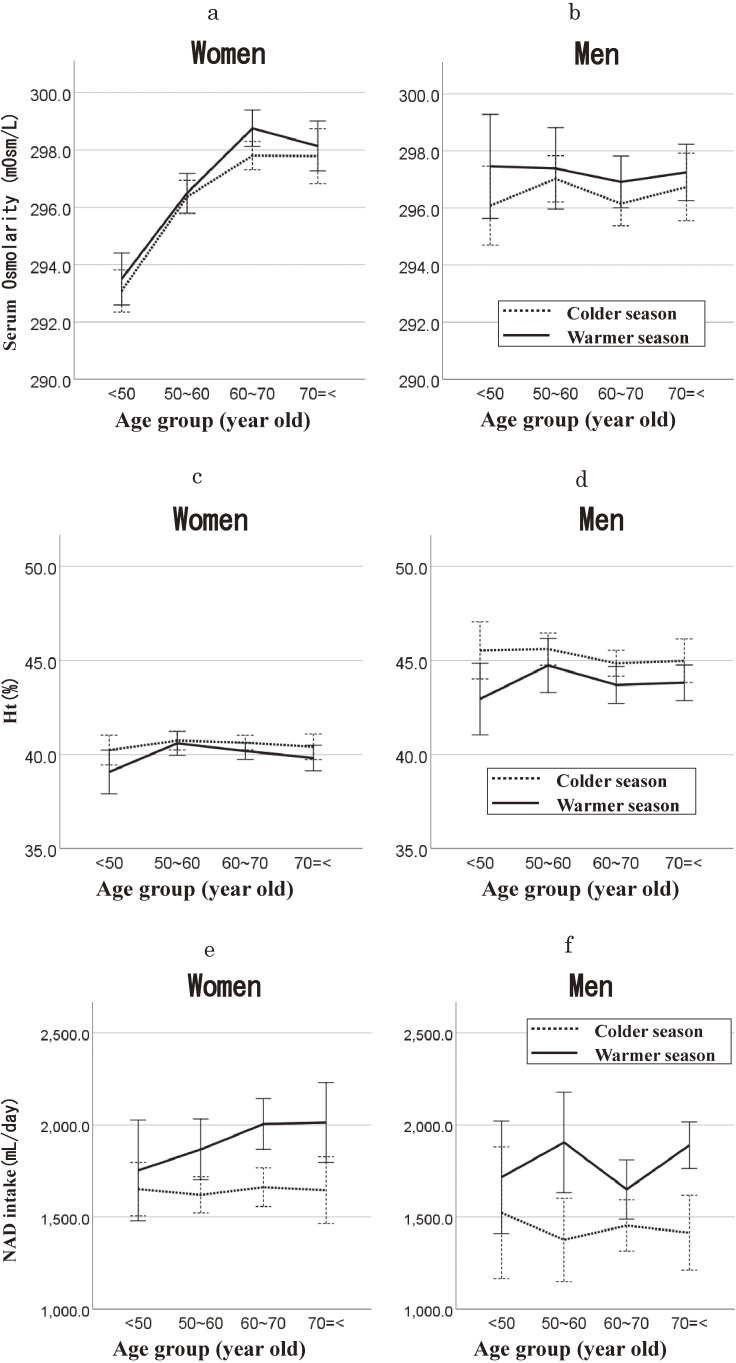
Seasonal differences in serum osmolarity, hematocrit, and non-alcohol drink intake, stratified by age group. The warmer season; from May to October, and the colder season; from November to April. The solid line represents the warmer season, and the dashed line represents the colder season. Mean serum osmolarity with error bars (95% confidence intervals) in women (a) and in men (b). Mean hematocrit with error bars (95% confidence intervals) in women (c) and in men (d). Mean non-alcohol drink intake (mL/day) with error bars (95% confidence intervals) in women (e) and in men (f).

Line graphs illustrating the relationship between age groups and serum osmolarity, hematocrit, and NAD intake for both sexes are presented in Fig. 1. Serum osmolarity exhibited a relative decrease in the colder season (Fig. 1a for women, Fig. 1b for men). Conversely, hematocrit showed a relative increase (Fig. 1c for women, Fig. 1d for men), and NAD intake was lower (Fig. 1e for women, Fig. 1g for men) compared to the warmer season across all age groups. Figure 1a shows that serum osmolarity tended to be higher in older age groups than younger groups for women in each season. However, this trend was not observed for men, as depicted in Fig. 1b. In contrast, the association between hematocrit and age group was not evident, as shown in Fig. 1c for women and Fig. 1d for men. Older women tended to consume more NAD than younger women in the warmer season, although age-associated differences were less apparent in the colder season (Fig. 1e). This trend was less evident in men (Fig. 1f).

We used multivariate analyses to evaluate an independent association of the serum osmolarity, hematocrit, and NAD intake with relevant factors (Table 2). The colder season was negatively associated with serum osmolarity and NAD intake but positively associated with hematocrit. Age was independently associated with serum osmolarity but not with Ht and NAD intake. Daily NAD intake was not associated with serum osmolarity and Ht. Seasonal variation of SBP was not observed, but its age-dependent increase was (Table 2d). Daily NAD intake was not associated with SBP. Similar findings were also observed in DBP (data not shown).

**Table 2:** Multivariate linear regression analyses on serum osmolarity, hematocrit, NAD intake, and systolic blood pressure

**a. Serum osmolarity**				

	**Coefficient**	**95% CI**	**Standardized** **coefficient**	**p value**
Colder/Warmer seasons	−0.619	(−1.054, −0.183)	−0.087	0.005
Women/Men	−1.152	(−1.924, −0.379)	−0.149	0.004
Age (y.o.)	0.132	(0.102, 0.162)	0.322	<0.001
Height (cm)	−0.020	(−0.065, 0.025)	−0.044	n.s.
BodyWeight (Kg)	0.037	(0.006, 0.069)	0.104	0.020
SBP	−0.010	(−0.035, 0.015)	−0.046	n.s.
DBP	0.025	(−0.013, 0.062)	0.073	n.s.
Ht. (%)	0.079	(0.003, 0.154)	0.077	0.040
NAD intake (mL/day)	0.000	(0.000, 0.000)	−0.026	n.s.
Hypertension	−0.357	(−1.119, 0.405)	−0.034	n.s.
Dyslipidemia	0.353	(−0.075, 0.781)	0.050	n.s.
Diabetes	0.473	(−1.001, 1.947)	0.019	n.s.
Current drinker	−0.263	(−0.718, 0.191)	−0.037	n.s.
Current smoker	0.386	(−0.674, 1.446)	0.022	n.s.
Sleeping hours	−0.099	(−0.310, 0.112)	−0.028	n.s.
Walking habit	0.015	(−0.445, 0.475)	0.002	n.s.
Living alone	0.062	(−0.642, 0.766)	0.005	n.s.

**b. Ht.**				

	**Coefficient**	**95% CI**	**Standardized** **coefficient**	**p value**
Colder/Warmer seasons	0.674	(0.315, 1.033)	0.096	<0.001
Women/Men	3.487	(2.883, 4.091)	0.462	<0.001
Age (y.o.)	−0.018	(−0.044, 0.007)	−0.045	n.s.
Height (cm)	−0.025	(−0.062, 0.013)	−0.055	n.s.
BodyWeight (Kg)	0.050	(0.024, 0.076)	0.144	<0.001
SBP	0.002	(−0.019, 0.023)	0.012	n.s.
DBP	0.060	(0.029, 0.091)	0.182	<0.001
Serum Osmolarity (mOsm/L)	0.054	(0.002, 0.105)	0.055	0.040
NAD intake (mL/day)	0.000	(0.000, 0.000)	0.021	n.s.
Hypertension	−0.481	(−1.111, 0.148)	−0.046	n.s.
Dyslipidemia	0.674	(0.322, 1.025)	0.097	<0.001
Diabetes	0.882	(−0.336, 2.100)	0.036	n.s.
Current drinker	−0.084	(−0.460, 0.293)	−0.012	n.s.
Current smoker	0.396	(−0.481, 1.272)	0.023	n.s.
Sleeping hours	0.186	(0.012, 0.361)	0.054	0.036
Walking habit	0.247	(−0.133, 0.627)	0.035	n.s.
Living alone	0.154	(−0.428, 0.737)	0.013	n.s.

**c. NAD intake**				

	**Coefficient**	**95% CI**	**Standardized** **coefficient**	**p value**
Colder/Warmer seasons	−306.553	(−390.5, −222.6)	−0.222	<0.001
Women/Men	−300.672	(−452.4, −148.9)	−0.202	<0.001
Age (y.o.)	3.352	(−2.726, 9.43)	0.042	n.s.
Height (cm)	−3.001	(−11.92, 5.919)	−0.034	n.s.
BodyWeight (Kg)	9.335	(3.125, 15.55)	0.135	0.003
SBP	−0.12	(−5.122, 4.882)	−0.003	n.s.
DBP	−2.988	(−10.43, 4.455)	−0.046	n.s.
Serum Osmolarity (mOsm/L)	−5.124	(−17.4, 7.153)	−0.027	n.s.
Ht. (%)	6.106	(−8.74, 20.95)	0.031	n.s.
Hypertension	−7.686	(−157.9, 142.5)	−0.004	n.s.
Dyslipidemia	−60.487	(−144.8, 23.82)	−0.044	n.s.
Diabetes	133.414	(−156.9, 423.7)	0.028	n.s.
Current drinker	−64.29	(−153.9, 25.31)	−0.047	n.s.
Current smoker	58.673	(−150.2, 267.6)	0.017	n.s.
Sleeping hours	−9.01	(−50.6, 32.58)	−0.013	n.s.
Walking habit	−141.431	(−231.6, −51.28)	−0.102	0.002
Living alone	186.627	(48.33, 324.9)	0.083	0.008

**d. SBP**				

	**Coefficient**	**95% CI**	**Standardized** **coefficient**	**p value**
Colder/Warmer seasons	−0.421	(−1.49, 0.651)	−0.013	n.s.
Women/Men	−1.648	(−3.55, 0.255)	−0.047	n.s.
Age (y.o.)	0.249	(0.175, 0.323)	0.133	<0.001
Height (cm)	−0.185	(−0.3, −0.07)	−0.088	0.001
BodyWeight (Kg)	0.038	(−0.04, 0.116)	0.023	n.s.
DBP	1.113	(1.051, 1.175)	0.728	<0.001
Serum Osmolarity (mOsm/L)	−0.061	(−0.21, 0.092)	−0.013	n.s.
NAD intake (mL/day)	<−0.001	(−0, 0.001)	−0.001	n.s.
Ht. (%)	0.022	(−0.16, 0.207)	0.005	n.s.
Hypertension	9.382	(7.604, 11.16)	0.194	<0.001
Dyslipidemia	1.082	(0.032, 2.131)	0.033	0.043
Diabetes	0.505	(−3.12, 4.124)	0.004	n.s.
Current drinker	0.219	(−0.9, 1.337)	0.007	n.s.
Current smoker	0.607	(−2, 3.21)	0.008	n.s.
Sleeping hours	0.409	(−0.11, 0.927)	0.026	n.s.
Walking habit	−0.104	(−1.23, 1.025)	−0.003	n.s.
Living alone	−0.184	(−1.91, 1.546)	−0.003	n.s.

SBP; systolic blood pressure, DBP; diastolic blood pressure, NAD; non-alcohol drink. Walking habit; >=2 days/a week, for >=30 min. n.s.; not significant (p >= 0.05). Warmer seasons; from May to October, Colder seasons; from November to April.

Coefficient; partial regression coefficient, Standardized coefficient; standardized partial regression coefficient.

## Discussion

The present study demonstrated that the colder season was negatively associated with serum osmolarity and NAD intake but positively associated with hematocrit in healthy subjects dwelling in one of the urban areas in Japan, using multivariate analyses. These findings suggests that intra-vascular volume depletion is more likely in the colder season despite lower serum osmolarity compared to the warmer season. Moreover, age dependent increase in serum osmolarity was observed, but not in Ht and NAD intake.

According to a global systematic review, when dehydration was defined as a serum osmolarity of 300 mOsm/L or higher, the prevalence of dehydration among non-hospitalized participants aged 65 and older was reported to be 24% [35]. Our results are consistent with this, as it was 27.2% when limited to participants aged 65 and older in the present study. Our results on seasonal fluctuation of serum osmolarity and Ht are also not contradictory to previous studies. The findings that the serum osmolarity was higher in the warmer season than in the colder season were consistent with another study observing elderly Japanese individuals [12]. The results that Ht was higher during the colder season is also consistent with previous studies [21, 36, 37]. Considering that serum osmolarity is used as a definitive diagnosis for water-loss dehydration [19] and hematocrit can be influenced by intra-vascular volume depletion [20], our findings suggest that intra-vascular volume depletion is more likely to occur in the colder season, while water-loss dehydration is more likely to occur in the warmer season. In cold environments, urine production increases for thermoregulation, thirst sensation decreases, and respiratory water loss is more likely due to dry air. These factors suggest a greater likelihood of decreased vascular fluid in the colder season.

Ht is thought to be a principal determinant of blood viscosity [21]. Beyond elevated blood viscosity from intra-vascular volume depletion [20], multiple factors contribute to ischemic events in colder season, such as blood pressure, vasoconstriction, and arterial fibrillation. When exposed to cold, the body reacts by increasing sympathetic nerve activity, which can reduce the diameter of arteries and increase peripheral resistance. Therefore, narrowing of blood vessels as well as increases blood pressure occur during cold seasons [13, 14, 38, 39]. These factors, such as vasoconstriction, elevates blood pressure, and increase in heart rate induced by increasing sympathetic nerve activity, are known to increase the risk of atrial fibrillation, particularly in susceptible individuals such as the elderly or those with pre-existing cardiovascular conditions [15, 16, 40]. This increase in the narrowing of blood vessels and the frequency of atrial fibrillation during winter not only raises the risk of ischemic diseases, but also, when combined with the rise in blood viscosity due to intra-vascular volume depletion, likely further elevates the risk of ischemic events during the cold season.

The present study also demonstrated increased serum osmolarity associated with aging, consistent with decreased body water content in older individuals [41]. On the other hand, no significant age-related changes were observed in hematocrit levels. Based on these findings, it can be inferred that elderly individuals are more prone to experiencing both water-loss dehydration and intra-vascular volume depletion. This condition, reduced body water but maintained Ht in older people, may decrease the margin for buffering intravascular fluid in colder season. Furthermore, considering that there was no statistically significant increase in NAD intake with age, it is speculated that older individuals may be more prone to inadequate intravascular fluid supply, or intra-vascular volume depletion, during colder season compared to warmer season. Therefore, it is reasonable to consider that these conditions contribute to the increased risk of ischemic diseases during colder season in the elderly.

This study has several limitations and strengths. The limitations are as follows: First, the cross-sectional design used in this study is limited in establishing causal relationships between the variables studied. As the data were collected at a single time point, we cannot determine the temporal sequence of events, and therefore, we cannot infer causality. Second, the study categorized warmer and colder seasons based on the surveyed month. However, seasonal transitions may vary between years, and the selected months may not precisely represent consistent weather patterns for each season. Third, all blood samples were obtained in the morning after fasting for at least 10 hours. This sampling style might have influenced the results the association between NAD intake and serum osmolarity as well as Ht. Therefore, the effect of daily NAD intake on protecting dehydration was not clear from the results in the present study. Fourth, this study did not specifically investigate the relationship between hydration status and vascular events, therefore, further research would be necessary to explore this topic in depth. Despite these limitations, the current study provides valuable insights into the association between seasonal variations, dehydration markers, and age in the study population. Further research with longitudinal designs and a more diverse population is needed to validate and expand upon these findings. On the other hand, the strengths are as follows: First, the study included 1009 participants comprising healthy individuals residing in Kobe, Japan. This large sample size enhances the generalizability of the findings to diverse populations. Finally, participants were analyzed using multivariate analysis to explore how seasonal changes affect serum osmolarity, hematocrit, and NAD intake, providing a thorough understanding of physiological impacts.

In conclusion, this study suggests that intra-vascular volume depletion is more likely in the colder season despite lower serum osmolarity compared to the warmer season. Furthermore, given that there is no age-related increase in water intake during winter, despite the age-related increase in serum osmolarity, water intake may be insufficient to adequately replenish body water, which can work as a buffer for vascular water, and prevent intra-vascular volume depletion in elderly individuals during this season. Usually, dehydration is well-known and often associated with heat-related illnesses during the hot summer months, and water intake during winter has not been adequately addressed. So, our findings emphasize the need for targeted interventions to address winter dehydration, especially among older adults. Implementing practical strategies to raise awareness about the importance of hydration during winter is essential to safeguard vulnerable populations’ well-being.

## Declarations

### Availability of data and materials

The most relevant data are within the paper. Raw data cannot be made publicly available, as study participants did not consent to have their information freely accessible.

Based on these consents, the Ethics Committee of the Institute of Biomedical Research and Innovation at the Kobe Biomedical Innovation Cluster (approval no. 10-20) and Keio University School of Medicine inhibit any public data sharing because data contain potentially identifying or sensitive disease information. Data accession requests may be sent to the administration of the Ethics Committees. The data will be shared after a review of the purpose and with permission from the ethics committees. Data requests can be made to the corresponding author.
